# Fecal Microbiota Transplantation Using Upper Gastrointestinal Tract for the Treatment of Refractory or Severe Complicated* Clostridium difficile* Infection in Elderly Patients in Poor Medical Condition: The First Study in an Asian Country

**DOI:** 10.1155/2016/2687605

**Published:** 2016-04-05

**Authors:** Tae-Geun Gweon, Jinsu Kim, Chul-Hyun Lim, Jae Myung Park, Dong-Gun Lee, In Seok Lee, Young-Seok Cho, Sang Woo Kim, Myung-Gyu Choi

**Affiliations:** ^1^Division of Gastroenterology, Department of Internal Medicine, Seoul St. Mary's Hospital, College of Medicine, The Catholic University of Korea, 222 Banpodaero, Seocho-gu, Seoul 137-070, Republic of Korea; ^2^Division of Infectious Diseases, Department of Internal Medicine, Seoul St. Mary's Hospital, College of Medicine, The Catholic University of Korea, 222 Banpodaero, Seocho-gu, Seoul 137-070, Republic of Korea

## Abstract

*Background and Aims*. Fecal microbiota transplantation (FMT) is a highly effective treatment option for refractory* Clostridium difficile* infection (CDI). FMT may be challenging in patients with a low performance status, because of their poor medical condition. The aims of this study were to describe our experience treating patients in poor medical condition with refractory or severe complicated CDI using FMT via the upper GI tract route.* Methods*. This study was a retrospective review of seven elderly patients with refractory or severe complicated CDI and a poor medical condition who were treated with FMT through the upper GI tract route from May 2012 through August 2013. The outcomes studied included the cure rate of CDI and adverse events.* Results*. Of these seven patients who received FMT via the upper GI tract route, all patients were cured. During the 11-month follow-up period, CDI recurrence was observed in two patients; rescue FMT was performed in these patients, which led to a full cure. Vomiting was observed in two patients.* Conclusions*. FMT via the upper gastrointestinal tract route may be effective for the treatment of refractory or severe complicated CDI in patients with a low performance status. Physicians should be aware of adverse events, especially vomiting.

## 1. Introduction

The incidence and severity of* Clostridium difficile* infection (CDI) have been increasing [1, 2]. Fecal microbiota transplantation (FMT) is an effective treatment modality for recurrent or refractory CDI. The therapeutic efficacy of FMT for the treatment of refractory CDI is >90% [3–5].

FMT can be performed via either the upper gastrointestinal (GI) tract route or the lower GI tract route. A systematic review reported that three out of four FMT procedures were performed via colonoscopy [6]. To date, no study has compared the therapeutic efficacy of CDI according to the infusion route. However, when FMT is performed using the upper GI tract route, the foul odor of the fecal suspension may cause discomfort, nausea, and vomiting in patients. This might contribute to the choice of the lower GI tract by physicians as an infusion route.

In FMT via the lower GI tract route, the fecal suspension is infused using colonoscopy or retention enema [6]. Although there is no guideline regarding the retention time of the fecal suspension when FMT is performed via this route, the fecal suspension should be retained in the colon as long as possible. One study reported that patients were asked to avoid defecation for 30–45 min [7].

Old age and severe underlying comorbidities are risk factors for CDI and predictable risk factors for CDI recurrence [8, 9]. Because these patients cannot retain the fecal suspension sufficiently, FMT via the lower GI tract route may be challenging in this group of individuals. Therefore, in this subset of patients, FMT may be performed via the upper GI tract route. The aims of this study were to describe our experience treating 7 patients in poor medical condition with refractory or severe complicated CDI using FMT via the upper GI tract route.

## 2. Patients and Methods

### 2.1. Study Participants and Assessment

This study was a retrospective review of seven elderly patients with refractory or severe complicated CDI and a poor medical condition who were treated with FMT through the upper GI tract route at Seoul St. Mary's Hospital and Uijeongbu St. Mary's Hospital, Republic of Korea, from May 2012 through August 2013. The demographic characteristics, the characteristics of CDI, the clinical outcomes of the study participants and adverse events related to FMT were investigated. Patients' performance status was evaluated using the Karnofsky Performance Status (KPS) score which runs from 0 to 100 (Table 1) [10]. Patients' comorbidities were recorded using the Charlson Comorbidity Index score [11]. A total of 22 conditions were assigned with a score of 1, 2, 3, or 6. Points were assigned to each condition as follows: 1, myocardial infarct, congestive heart failure, peripheral vascular disease, dementia, cerebrovascular disease, chronic lung disease, connective tissue disease, ulcer, chronic liver disease, and diabetes; 2, hemiplegia, moderate or severe kidney disease, diabetes with end organ damage, tumor, leukemia, and lymphoma; 3, moderate or severe liver disease; 6, malignant tumor, metastasis, and acquired immune deficiency syndrome. The mental status and cognitive functions of patients were also assessed. WBC count and serum creatinine levels were recorded at the time of diagnosis of CDI, before FMT.

CDI was defined as a combination of a toxigenic stool culture and diarrhea ≥3/day [12]. The stool culture (chromID* C. difficile*; bioMérieux, Marcy l'Etoile, France) and a toxin assay using enzyme immunoassay (Wampole Tox A/B Quik Chek; Alere, Orlando, FL, USA) and polymerase chain reaction for the detection of toxin genes (*tcdA*,* tcdB*,* cdtA*, and* cdtB*) were performed. Refractory CDI was defined as an unresponsiveness to more than 14 days of a conventional therapy that included oral vancomycin. Severe, complicated CDI was defined as a combination of CDI and presence of abdominal distension, documented bowel dilatation on abdominal CT scan, and hemodynamic instability [12, 13]. Resolution of CDI was defined as the status in which all of the following criteria were met: (1) cessation of diarrhea 1-2 days after FMT and (2) negative conversion of a toxigenic stool culture. Recurrence was defined as the presence of the criteria used to define CDI at least 2 weeks after its resolution. Poor medical condition was defined as (1) KPS score ≤40, which warrants hospitalization and special treatment and/or (2) multiple comorbidities. This study's protocol was approved by the Institute Review Board of Seoul St. Mary's Hospital and of Uijeongbu St. Mary's Hospital.

### 2.2. Fecal Microbiota Transplantation

The donor-stool source was a family member or an unrelated healthy donor. Before FMT, we asked the patient's family to select the stool donor. Subsequently, the donor's medical history as well as stool and blood samples were screened. The hepatitis B surface antigen, the hepatitis C virus antibody, and the human immunodeficiency virus were checked, and a serological test for syphilis was performed. The test of the donor's stool included a white blood cell count, an ovum and parasite,* Salmonella* culture, and* C. difficile* toxin. The donor had not used antibiotics within the past year and had no history of chemotherapy. The donor's stool (>50 g) was collected within 24 h before FMT. Stool and normal saline (1 : 3) were placed in a blender (NJM-9060; NUC Electronics, Daegu, Korea) and ground for 3 min. The fecal suspension was passed through a stainless steel tea strainer, to remove large particles. Colonoscopy was performed in all patients before FMT, to detect pseudomembranous colitis (PMC). However, FMT was performed via the upper GI tract route, as the patients were not able to retain the fecal suspension because of their poor medical condition. The fecal suspension was infused using upper endoscopy or a percutaneous endoscopic gastrostomy (PEG) tube with a 50 mL syringe. A sedative was administered to patients whose vital signs were stable. The patients were kept in a 45° upright position for 4 h after FMT. Written informed consent was given by the patient or their family before FMT.

## 3. Results

### 3.1. Patient Characteristics

The medical records of seven patients were reviewed. There was no previous FMT history in six of the seven patients. One patient received two courses of FMT via colonoscopy, which were not successful. The demographic and clinical data of the patients are shown in Table 2. All patients were immobilized and were treated as inpatients. The mean age was 75.6 years, and all patients had multiple comorbidities. The mean score on the KPS scale was 17.1 (range, 10–20). The median Charlson Comorbidity Index score was 3 (range, 1–14). One patient was intubated for the treatment of acute respiratory distress syndrome with sedation. Five of the seven patients had pneumonia as the index infection. The mean number of diarrhea events was 5.4/day, PMC was observed in five patients (71.4%), and the mean number of CDI episodes was 2.86. Five patients received FMT for refractory CDI, and two received FMT for severe, complicated CDI.

### 3.2. FMT and Post-FMT Data

Seven patients who received FMT via the upper GI tract route were initially cured that have met with the criteria of CDI resolution. One patient who had received two courses of FMT using colonoscopy before receiving FMT via the upper GI tract route exhibited symptoms that were compatible with severe, complicated CDI. The previous two courses of FMT, which were delivered via colonoscopy, were incomplete, as cecal intubation could not be performed because of severe abdominal distension and because the patient was not able to retain the fecal suspension sufficiently. CDI was cured in this patient after the FMT using upper endoscopy [14]. During the 11 months (mean) of the follow-up period (range, 5–17 months), recurrence was observed in two patients and occurred 90 and 130 days after FMT, respectively.* C. difficile*-provocative antibiotics were prescribed after FMT to each of these patients for 45 and 90 days, respectively. Rescue FMT was performed in patients with recurrence via upper endoscopy using the same donor. Recurrent CDI was cured, and recurrence was not observed 6 and 9 months after rescue FMT. Thus, the upper GI route of FMT was successful in all of the 7 patients, mostly in 1 session. The total number of episodes of CDI, including recurrence, was nine. Nine sessions of FMT were performed. The infusion route of the fecal suspension was as follows: upper endoscopy, eight sessions; PEG tube, one session. The mean amount of stool used was 91.2 g (range, 50–150 g). Five sessions of FMT (55.6%) were performed using a stool sample from a family donor. Sedatives were used in eight cases of FMT (88.9%).

### 3.3. Adverse Events

Among the seven patients included in the study, two vomited. One patient vomited 30 min after FMT and the other patient vomited 3 h after FMT. Although the amount of vomitus could not be measured, it was not significant. Other adverse events, including aspiration pneumonia, were not observed in these two patients.

## 4. Discussion

In this study, we treated refractory or severe complicated CDI in patients with a poor medical condition via FMT using the upper GI tract route; all patients were cured after FMT. Even though the indication of FMT has been increasing recently [15–17], there are few published articles on FMT in Asian countries [14, 18, 19]. This might be associated with the lower incidence of refractory CDI in these countries. A comprehensive single-center study of CDI performed in the Republic of Korea reported that the incidence of refractory CDI was 0.7% (2/320) in 2011 [20]. This lower incidence of refractory CDI is caused by the low prevalence of the hypervirulent strain B1/NAP1/027 in the Republic of Korea [21]. CDI is more prevalent in Western countries than it is in Asian countries. The incidence of hospital-acquired CDI in the Republic of Korea was reported as being up to 9.1 cases/10,000 patient hospital days [20, 21], which is lower than that observed in North America (28.1 cases/10,000 patient hospital days) [22]. Furthermore, the proportion of community-acquired CDI in North America was reported to be as high as 40% [23, 24], versus 3.4% in the Republic of Korea [18]. Well-known risk factors for CDI, including old age, hospitalization, and comorbidities, are absent in community-acquired CDI [25]. Antimicrobial exposure, use of proton-pump inhibitors, and* C. difficile* transmission in an outpatient setting are important risk factors for community-acquired CDI [25, 26]. B1/NAP1/027 was the most common (21.7%) strain isolated in community-acquired CDI [26]. This finding implies that CDI may be unresponsive to conventional treatment, even in a community setting, in North America. Most FMT procedures are performed in an outpatient manner in North America [4, 15, 27]. To our knowledge, there are no reports of refractory CDI in patients with community-acquired CDI in Asian countries. In these countries, refractory CDI that is unresponsive to conventional treatment might be complicated in patients with poor medical condition.

In our study, the medical condition of patients was poor because of old age, low performance status, or the presence of multiple comorbidities. FMT in patients with poor medical condition can be challenging. To minimize the risk of procedure-related complications, the procedure time should be shortened as much as possible. FMT via the upper GI tract route has some advantages compared with FMT performed via colonoscopy: (1) a shorter procedure time, (2) no need for bowel cleansing, and (3) a longer retention time in the large bowel, regardless of the patient's medical condition and consciousness. All FMT sessions were performed using the upper GI tract route. Only one patient received FMT via colonoscopy before the FMT using the upper GI tract. The two previous FMT sessions were partially effective, and abdominal distension and the number of diarrhea events were slightly decreased. However, the procedure was incomplete because cecal intubation was not performed and the patient was not able to retain the fecal suspension sufficiently. Diarrhea was improved, but not resolved. Drowsy patients cannot retain the fecal suspension sufficiently; therefore, we performed FMT using upper endoscopy. CDI was completely resolved after FMT via the upper GI tract route.

Recurrence of CDI was observed in two patients, 90 and 130 days after FMT, respectively. Continuous use of* C. difficile*-provocative antibiotics and comorbid conditions are well-known risk factors for CDI recurrence [28, 29].* C. difficile*-provocative antibiotics were prescribed in patients with recurrence. Moreover, patients had multiple comorbidities. Because the time between FMT and recurrence was not short, FMT efficacy was not associated with recurrence; rather, recurrence seemed to be caused by the medical condition of the patients.

Patients with a poor medical condition have a high risk of adverse events after FMT [15]. In the current study, vomiting was observed in two of seven patients (28.6%), who vomited when they were sitting in an upright position after FMT. Vomiting and nausea are important adverse events after FMT performed via the upper GI tract route [5, 15]. Aspiration pneumonia after FMT may cause death [15]. Thus, FMT via the upper GI tract route should be performed with caution, and close monitoring of nausea and vomiting is necessary to prevent aspiration pneumonia in elderly patients with a poor medical condition. To prevent vomiting related to FMT delivered via the upper GI tract route, the fecal suspension may be infused into the proximal jejunum using push enteroscopy or balloon-assisted enteroscopy.

This work was the first study of FMT for the treatment of refractory or severe complicated CDI conducted in an Asian country. The limitations of the current study included its small sample size and retrospective design. Although this study was retrospective, the authors collected the data prospectively. We suggest that FMT via the upper GI tract route is an effective option for the treatment of refractory or severe complicated CDI in patients with old age and a poor medical condition who are not eligible for FMT through the lower GI tract route. Moreover, this method can be tried as a rescue or alternative treatment option when FMT using colonoscopy fails or is incomplete. Further protocol improvement is needed to reduce procedure-related adverse events.

## Figures and Tables

**Table 1:** Karnofsky Performance Status score.

Score	Criteria
100	Normal; no complaints; no evidence of disease.
90	Able to carry on normal activity; minor signs or symptoms of disease.
80	Normal activity with effort; some signs or symptoms of disease.
70	Cares for self; unable to carry on normal activity or to do active work.
60	Requires occasional assistance but is able to care for most of their personal needs.
50	Requires considerable assistance and frequent medical care.
40	Disabled; requires special care and assistance.
30	Severely disabled; hospital admission is indicated although death is not imminent.
20	Very sick; hospital admission necessary; active supportive treatment necessary.
10	Moribund; fatal processes progressing rapidly.
0	Dead.

**Table 2:** Pre-FMT and post-FMT data of the patients.

Patient number	Age	Sex	K-P scale	Mental status	Cognition	Index infection	CCIs	Number of diarrhea per day	WBC count (cell/mcL)	Cr (mg/dL)	PMC	Number of CDI before FMT	Days from first CDI diagnosis to FMT	Days of last course of antibiotic treatment for CDI	Indication of FMT	Adverse events
1	83	Male	20	Alert	Impaired	Pneumonia	2	5	4,200	0.42	Yes	1	15	15	Refractory CDI	None
2^∧^	87	Male	20	Alert	Intact	Pneumonia	8	4	3,980	0.86	Yes	5	149	41	Refractory CDI	Vomiting
3^*∗*∧^	74	Male	10	Drowsy	Impaired	Pneumonia	14	10	5,200	2.57	Yes	2	29	10	Severe, complicated CDI	None
4	55	Male	20	Stupor	Impaired	Infectious colitis	3	5	6,240	0.68	No	5	486	37	Refractory CDI	None
5	75	Female	20	Alert	Intact	Urinary tract infection	3	5	18,830	2.3	No	4	459	7	Severe, complicated CDI	Vomiting
6	72	Male	20	Alert	Impaired	Pneumonia	6	4	12,350	0.97	Yes	2	237	27	Refractory CDI	None
7	83	Male	10	Sedated	Uncheckable	Pneumonia	1	5	8,440	0.64	Yes	1	37	37	Refractory CDI	None

FMT, fecal microbiota transplantation; K-P scale, Karnofsky Performance scale; CCIs, Charlson Comorbidity Index score; WBC, white blood cell; Cr, creatinine; PMC, pseudomembranous colitis; CDI, *C. difficile* infection.

^∧^Patients who had recurrence after FMT.

^*∗*^Patient who received 2 sessions of FMT using colonoscopy before FMT using upper endoscopy.
